# Antiretroviral pre-exposure prophylaxis implementation in the United States: a work in progress

**DOI:** 10.7448/IAS.18.4.19980

**Published:** 2015-07-20

**Authors:** Kenneth H Mayer, Sybil Hosek, Stephanie Cohen, Albert Liu, Jim Pickett, Mitchell Warren, Douglas Krakower, Robert Grant

**Affiliations:** 1 The Fenway Institute, Fenway Health, Boston, MA, USA; 2 Beth Israel Deaconess Medical Center, Boston, MA, USA; 3 Harvard Medical School, Boston, MA, USA; 4 Stroger Hospital of Cook County/CORE Center, Chicago, IL, USA; 5 San Francisco Department of Health, San Francisco, CA, USA; 6 AIDS Foundation of Chicago, Chicago, IL, USA; 7 AIDS Vaccine Advocacy Coalition, New York, NY, USA; 8 Gladstone Institute, University of California, San Francisco, CA, USA

**Keywords:** PrEP, pre-exposure prophylaxis, tenofovir-emtricitabine

## Abstract

**Introduction:**

After the initial approval of the use of tenofovir disoproxil fumarate-emtricitabine (TDF/FTC) by the US Food and Drug Administration in 2012 for anti-HIV pre-exposure prophylaxis (PrEP), uptake was initially limited, but more recent community surveys and expert opinion suggest wider acceptance in some key populations.

**Discussion:**

Demonstration projects are underway to determine the best practices in the United States to identify at-risk individuals in primary care and sexually transmitted disease clinics who could benefit from PrEP. Studies of PrEP in combination with behavioural interventions are being evaluated. Studies to evaluate the use of PrEP by HIV-uninfected women in HIV-discordant couples interested in safe conception are also getting underway. The optimal deployment of PrEP as part of a comprehensive national HIV/AIDS strategy in the United States has been limited by lack of knowledge among some at-risk people and by some medical providers indicating that they do not feel sufficiently knowledgeable and comfortable in prescribing PrEP. Studies are underway to determine how to assist busy clinicians to determine which of their patients could benefit from PrEP. Although most federal health insurance programmes will cover most of the costs associated with PrEP, underinsured patients in states that have not enacted health reform face additional challenges in paying for PrEP medication and appropriate clinical monitoring.

**Conclusions:**

PrEP implementation in the United States is a work in progress, with increasing awareness and uptake among some individuals in key populations.

## Introduction

### From clinical trials to PrEP approval

In 2010 to 2011, the first data from pre-exposure prophylaxis (PrEP) efficacy trials were reported, demonstrating that oral tenofovir disoproxil fumarate-emtricitabine (TDF/FTC) could protect against HIV acquisition. The first demonstration of PrEP efficacy was in the iPrEx trial, which enrolled men who have sex with men (MSM) in Latin America, the United States, South Africa and Thailand [1]. This study, coupled with the demonstration of efficacy in African heterosexuals [2, 3], led to the approval of the use of TDF/FTC for chemoprophylaxis by the Food and Drug Administration in the summer of 2012 and to formal PrEP guidelines issued by the US Centers of Disease Control and Prevention (CDC) [4].

Despite the initial reports of PrEP efficacy, concerns were raised because of the less-than-optimal adherence in iPrEx (approximately 51% had detectable drug levels in their blood) and two PrEP studies in African women that did not demonstrate protection [5, 6]. It was thought that PrEP might not produce a meaningful public health benefit because of “real-world” problems achieving optimal adherence. Fewer than 200 of the 2499 participants in iPrEx were American, so PrEP efficacy data in the United States were limited. However, subsequent American demonstration projects have suggested that, when individuals use open-label PrEP on a voluntary basis, adherence may be better, because users self-select to use PrEP to protect themselves against HIV (Table 1).

**Table 1:** Ongoing and planned PrEP trials and demonstration projects, as of November 2014

Trial/project	Sponsor/funder	Type/Category	Location	Population	Design/key questions	Status
**The Demo Project**	National Institute of Allergy and Infectious Diseases of the NIH	Demonstration Project	US (Miami, FL; San Francisco, CA; and Washington, DC)	MSM and transgender women	Assesses uptake, acceptability, safety and feasibility of once-daily TDF/FTC as PrEP in 600 MSM (300 in San Francisco; 200 in Miami; 100 in Washington)	Ongoing; expected completion date January 2015
**East Bay Consortium/CRUSH (Connecting Resources for Urban Sexual Health)**	California HIV/AIDS Research Program of the University of California	Demonstration Project	US (East Bay, CA)	Young MSM of colour	Testing and linking young MSM of colour to sexual health services; enhance engagement and retention for HIV-positive young MSM of colour; and retain HIV-negative young MSM of colour in sexual health services, including PrEP	Ongoing; started in December 2012
**LAC PATH PrEP Demo Project**	California HIV/AIDS Research Program of the University of California; LA County HIV & STD Program; Los Angeles Gay and Lesbian Center; OASIS Clinic; AIDS Project LA; UCLA	Demonstration Project	US (Los Angeles, CA)	MSM	Evaluates a customized prevention package that may include PrEP Enrolling 375 high-risk MSM and transgender women	Ongoing; expected completion date of May 2017
**California Collaborative Treatment Group Consortium/ALERT (Active Linkage, Engagement and Retention to Reduce HIV)**	California HIV/AIDS Research Program of the University of California, San Diego County HIV, STD, and Hepatitis Branch and the Long Beach Health and Human Services Agency	Demonstration Project	US (Long Beach, Los Angeles and San Diego, CA)	MSM	Evaluates whether a text messaging-based adherence intervention can improve adherence to the PrEP medication. Enrolling 400 high-risk MSM randomized to receive daily TDF/FTC as PrEP	Ongoing; expected completion date October 2015
**SPARK Project NYC**	HART and Callen-Lorde Community Health Center, funded by the National Institute on Alcohol Abuse and Alcoholism	Demonstration Project	US (New York)	MSM and transgender women	Evaluates a comprehensive prevention package that includes PrEP and examines social and behavioural factors associated with disparities in access to prevention and care services among gay, bisexual and other men who have sex with men that might impact PrEP implementation programs	Ongoing; started October 2013. Expected completion of July 2017
**Project PrEPare (Adolescents 18–22)** **Project PrEPare (Adolescents 15–17)**	Adolescent Medicine Trials Network for HIV/AIDS Interventions (ATN); funded by NICHD, NIDA, NIMH	Open-Label Demonstration Project and Phase II Safety Study	US (Baltimore; Boston; Bronx, NY; Chicago; Washington, DC; Denver; Detroit; Houston; Los Angeles; Memphis; Miami; New Orleans; Philadelphia; Tampa)	MSM MSM	Explores the safety, acceptability and feasibility of PrEP among young men who have sex with men (YMSM) who are at risk for HIV infection. Enrolling 300 HIV-uninfected YMSM	Ongoing, started November 2012; expected completion November 2015 Ongoing; expected completion March 2016
**HPTN 073**	HPTN; funded by NIAID/NIH	Open-Label Demonstration Project	US (Los Angeles, CA; Washington, DC; Chapel Hill, NC]	MSM	Assesses the initiation, acceptability, safety and feasibility of PrEP for Black MSM (BMSM); subset of participants will be recruited for qualitative interviews about PrEP facilitators and barriers. Enrolling a total of 225 participants	Ongoing; started July 2013
**CDC Foundation Demonstration Project**	Funding pending	Demonstration Project	US	MSM and heterosexual women	Proposed to evaluate real-world PrEP use in MSM and heterosexual women at risk of HIV infection in health clinic settings, potentially in 1200 participants	Start date pending funding
**iPrEx OLE**	Sponsored/funded by DAIDS/NIH, through a grant to the Gladstone Institutes.	Open-label extension	Brazil, Peru, Ecuador, South Africa, Thailand, US	MSM	Continuation of the iPrEx study designed to provide additional information about the safety of PrEP and the behaviour of people taking PrEP over a longer term	Completed. Results announced July 2014

*Source*: Donaldson E, Grant D and Warren M. Ongoing and Planned US PrEP Trials and Demonstration Projects. Available from: www.avac.org/prevention-option/prep/usdemoprojects [cited 2014 Dec 1].

## Demonstration projects

In order to assess the impact of PrEP after participants learned that it was effective, iPrEx participants were offered access to medication through an open-label extension (iPrEx OLE) protocol [7]. Approximately 65% of the original participants in iPrEx and 68% of participants in the Adolescent Trials Network (ATN) protocol ATN 082 [8] and an earlier CDC safety study [9] who were eligible participated in the iPrEx OLE study. Participants were asked to provide written informed consent and were offered PrEP or ongoing observation without medication at the start of the iPrEx OLE. All participants came in for HIV testing and counselling at quarterly intervals. Most of those (72%) who entered the iPrEx OLE study elected to start PrEP right away and 6% more started using PrEP sometime after enrolment. People were more likely to enrol in iPrEx OLE if they had a history of condomless anal intercourse and/or sexually transmitted disease (STD), suggesting that the Open Label Extension was attractive to those who might benefit most. The majority of participants who elected to use PrEP had detectable drug in the blood when periodically screened. Among participants whose drug levels were consistent with taking TDF/FTC four or more times a week, no seroconversions occurred, compared to an incidence rate of 2.6% for those iPrEx OLE participants who elected not to take PrEP.

The first post-iPrEx US PrEP demonstration project was conducted at STD clinics in San Francisco and Miami and a community health centre in Washington, DC [10, 11]. Six hundred individuals who were PrEP-naïve were recruited via local media and community venues. These individuals were asked to provide written informed consent prior to the initiation of PrEP, so their experiences could be carefully monitored over the course of the subsequent year. The majority of the participants were white, but 7.2% were black and 1.3% were transgender. The study found that there was a good deal of community interest in PrEP at the sites. Among 90 participants whose blood was sampled at week 24, 90% had tenofovir levels consistent with taking at least four doses per week (97% in Washington, DC, 93% in San Francisco and 81% in Miami). Pharmacological modelling studies suggest that these drug levels correlate with a high level of protection [12]. Other demonstration studies have gotten underway in Southern California, enrolling participants in STD clinics and HIV specialty care centres. One of the California studies has included behavioural counselling and drug-level assessment to enhance adherence [13]. Individuals whose drug levels were found to be low received additional counselling. However, there was little need for enhanced counselling, because the majority of participants were highly adherent.

Studies elsewhere are underway to develop other approaches to facilitate PrEP adherence. A team working at Boston's Fenway Health has tested PrEP support tools based on Lifesteps, an evidence-based protocol developed to improve adherence for HIV-infected individuals [14]. In a pilot study funded by the US National Institutes of Health (NIH), participants found the PrEP intervention, which includes four weekly counselling sessions delivered by a nurse, to be highly acceptable and 84% had drug levels consistent with daily PrEP use at six months [15]. Another study is evaluating the use of a mobile health (mHealth) strategy to support PrEP adherence [16] by adapting an SMS-based intervention previously shown to increase ARV adherence and virologic suppression rates in HIV-infected individuals [17]. Gilead Sciences, the developer of Truvada™, has supported several demonstration projects in the United States and elsewhere. PrEP demonstration studies are underway in several southern cities with high rates of new HIV infections, such as Houston, TX, and Jackson, MI, and smaller cities on the East Coast, such as Providence, RI.

## Focused population studies

Although internationally the iPrEx Study included a substantial number of younger MSM, there was limited enrolment of the most vulnerable youth in the United States, young black and Latino MSM. The ATN conducted a PrEP feasibility study in Chicago, which found that youth were interested in taking PrEP, but overall adherence was about 50% [8]. The ATN is now studying whether PrEP adherence in two parallel studies can be improved with either of two evidence-based HIV-prevention interventions, one individualized and the other a group intervention (ATN 110 for participants aged 18 to 22 years old and ATN 113 for those aged 15 to 17 years old). Almost half (49%) of the participants in ATN 110 are black, 27% are Latino and 5% are transgender women; 33% of the participants in ATN 113 are black and 49% Latino.

Although black MSM in the United States are disproportionately affected by HIV [18–21], they were not the primary focus of prior studies. Black MSM do not engage in condomless anal sex more often than other MSM, but, because of assortative mixing, poverty and other adverse social consequences, black MSM remain at great risk for HIV acquisition, and many would be appropriate candidates for PrEP [18–21]. The HPTN 073 study is a demonstration project that has recruited 225 black MSM in Washington, DC, North Carolina and Los Angeles. The study has enrolled 225 black MSM, who have been offered clinical monitoring and care coordination with or without PrEP. The coordinated clinical care is designed to address unmet social and structural needs (e.g. health insurance, stable housing) as well as behavioural health concerns (e.g. depression or substance use), any of which might impede PrEP adherence. The educational materials and counselling protocols have been culturally tailored by a team of black MSM researchers. The study is still underway but, encouragingly, the majority of enrolees chose to initiate PrEP.

Although more than a quarter of new US HIV infections occur in heterosexual women, identifying specific women who might most benefit from PrEP has been challenging [22]. Many women who become HIV-infected may be unaware of their partner's serostatus; because of power dynamics in their primary relationship, they may not be able to negotiate safer sex with their partners. Because black MSM are a minority among MSM, strategies designed to recruit those at greatest risk for HIV are straightforward, for example, focusing on social venues and media used by black MSM. Identifying at-risk women is more challenging, because there are millions of American women who live in high prevalence communities, but most are not likely to become HIV-infected by their primary partners. A recent study designed to assess the predictors of HIV incidence among at-risk women (HPTN 064, the “ISIS” study) found an annualized HIV incidence of 0.25% [23]. This low level of HIV incidence would make it very difficult to conduct an efficacy trial to evaluate the benefit of PrEP for high-risk US women, because thousands would need to be enrolled in order to demonstrate efficacy. Given the low HIV incidence in US women, concerns have been raised about the chronic use of PrEP (given costs and toxicities) to prevent the rare likelihood that individual women would become HIV-infected. However, women who are in an HIV-discordant relationship could clearly benefit from regular use of PrEP. Some have argued that if the HIV-infected partner initiates antiretroviral therapy and is stably virologically suppressed for at least six months, the female partner does not need to use PrEP. However, this strategy would rely on the woman having perfect knowledge that her partner was adherent and virologically undetectable. Particularly for couples that are contemplating having children, for whom an HIV transmission to the foetus or infant would be unacceptable, “PrEPception” may offer some major advantages. A multicentre group of women's health investigators are offering HIV-discordant couples a menu of options, including virologic suppression of the infected partner, PrEP for the HIV-negative female partner, as well as assisted reproduction. This study will not be powered to assess the efficacy of any one strategy but will provide invaluable insights about the acceptability of the different approaches to protecting child-bearing women who have HIV-infected partners.

Transgender people are highly affected by HIV [24]. Because a relatively low percent of iPrEx participants were transgender women, there are insufficient *data* regarding PrEP safety, acceptability and efficacy for them. The iPrEx OLE study found that TDF/FTC concentrations were, on average, lower among transgender women compared with MSM [7]. Although suboptimal medication adherence is thought to explain some of the differences, the possibility that drug-drug interactions of exogenous feminizing sex hormones could alter intercellular FTC or TDF concentrations is under study. Further studies of PrEP for transgender women are needed.

## Community responses

In a manner very analogous to the rollout of hormonal contraception a half century ago, the responses to the proof of efficacy of PrEP have been quite mixed [25]. Many gay and reproductive rights activists have applauded the advent of this new prevention option; however, other individuals have seen PrEP as a preventive intervention that is fraught with danger. Some American gay community leaders have issued ads and pronouncements expressing concerns about PrEP, unsubstantiated by available data. The concerns have included questions about whether PrEP will increase behavioural disinhibition, resulting in new HIV transmissions, whether it will promote the development of resistant strains of HIV, and/or increase rates of STDs. Concerns have also been raised regarding rates of toxicity of the medications, because side effects may be less acceptable for individuals who are otherwise healthy than for people at risk of developing AIDS without medication. In response to some of these criticisms, several organizations that focus on MSM health, sexual and reproductive health rights and community education have developed public information campaigns, often conducted on the internet, to correct misinformation (Table 2). These organizations include the AIDS Foundation of Chicago, the San Francisco AIDS Foundation, Fenway Health and Project Inform. These groups have also attempted to educate at-risk populations and providers through opinion pieces in local and national media. Most recently, the largest and longest running national coalition of community-based HIV/AIDS organizations – the AIDS United Public Policy Committee – issued a call to end the debate about PrEP and shift the national focus to scale-up of this intervention [26].

**Table 2:** Useful US-focused PrEP resources

Organization	Webpage
AIDS Foundation of Chicago	www.myprepexperience.blogspot.com/
Project Inform	www.projectinform.org/prep/
San Francisco AIDS Foundation	www.prepfacts.org
The Fenway Institute	www.thefenwayinstitute.org/prepinfo/
The US Centers for Disease Control and Prevention	www.cdc.gov/hiv/prevention/research/prep
The AIDS Vaccine Advocacy Coalition	www.avac.org

Several individuals who believe that wider access to PrEP will provide more options for individuals to make safer choices have noted that some of the controversy in the gay and mainstream media has actually increased the knowledge base of individuals who might benefit from PrEP. The dissemination of correct information remains extremely important: recent surveys in one of the larger social networking sites, Manhunt, found in early 2014 that only 3.1% of almost 9000 MSM respondents had used PrEP and that substantial numbers had not heard of it [27]. Additionally, a study by Gilead Sciences with one of the largest national retail pharmacy chains found that there were only around 2,500 individuals who had received PrEP, and about half of them were women [28]. More recent surveys and discussions with key opinion leaders suggest that PrEP utilization may be growing, but the scale of PrEP utilization among those who could benefit the most remains unclear.

## Provider issues

Several studies have found that one of the biggest barriers to the provision of PrEP are the reticence of health-care providers, some of whom have expressed concerns about behavioural disinhibition, risk compensation, costs and potential toxicities with PrEP [29–32], particularly in communities where the number of persons needing to be placed on PrEP may be high relative to the number of HIV infections averted. Given that many providers in general practice do not routinely ask their patients about their sexual orientation or gender identity [33, 34], conversations about the appropriateness of PrEP may not be easily undertaken. Several organizations, such as Fenway Health, have developed provider education campaigns, including monograph and webinars (www.lgbthealtheducation.org), that supply key PrEP information for providers and potential consumers.

Concerns have been raised that insurers would not pay for PrEP. Because the United States does not have an integrated health-care system and health is primarily regulated by the states, there have been a variety of responses by regulatory authorities to PrEP. For the most part, private insurance companies will cover the cost of PrEP, but, depending on the type of insurance an individual has, co-payments as high as $100 per month may be expected, thereby eliminating PrEP access for individuals who have modest incomes and inadequate insurance. Gilead Sciences maintains a patient assistance program, which has been beneficial to individuals with very limited economic means, but it has left gaps for others who have high co-payments, but whose salaries are above the threshold for these programs [35]. In states that have accepted the expansion of Medicaid, as part of the implementation of the Affordable Care Act, few residual barriers exist for support for PrEP implementation. In addition to the cost of the actual medication, which can be close to $15,000 per year if paid out of pocket, there are other attendant medical costs, since best practices mandate that PrEP users should be routinely counselled and tested for HIV and bacterial STDs on at least a quarterly basis, as well as having their renal function monitored.

## Conclusions about PrEP in the United States

Several studies are underway in the United States, as well as internationally, that may have an impact on how PrEP is delivered over the next few years [36]. In October 2014, the British PROUD open-label oral TDF/FTC PrEP demonstration project (www.proud.mrc.ac.uk/) determined that MSM assigned to receive PrEP had an 86% decrease in their risk of becoming HIV-infected compared to participants assigned to the waiting-list condition [37]. The importance of this study is that it is the first demonstration project to clearly show that real-world access to PrEP can significantly decrease HIV incidence in MSM.

Because maintenance of high levels of adherence has been a challenge for many earlier trial participants – as well as to address concerns that have been raised about resistance, cost and drug toxicity – studies are underway to assess whether more parsimonious dosing schedules may be protective. In the iPrEx Study, a retrospective analysis of drug levels found that individuals who took the medication at a frequency of approximately four times per week had a comparable level of protection to those who took the medication on a daily basis [13]. A study conducted in France and Quebec, iPERGAY, is a placebo-controlled trial evaluating pericoital oral TDF/FTC prophylaxis in MSM. This trial has found that MSM assigned to receive active medication were 86% less likely to become HIV-infected than those assigned to the placebo condition [38]. The US CDC and other public health authorities have not endorsed less-than-daily PrEP dosing at this point, because there are data from multiple trials supporting this approach, but as additional data regarding event-driven, pericoital oral PrEP become available, recommendations could change. Other studies that may influence how PrEP is prescribed include studies of different oral medications (e.g. maraviroc) and different delivery systems (e.g. injections and vaginal rings).

Although some of the key research showing the efficacy of PrEP was conducted in the United States, and US regulatory authorities have approved its use for at-risk individuals engaging in condomless sex, uptake has been slower than expected by some, given that about 50,000 Americans become HIV-infected annually. On the other hand, some innovations may take more than a decade to become more widely used. Some critics have raised concerns about the unintended consequences of PrEP use, including risk compensation, selection for drug resistance, unappreciated drug toxicity and cost. Despite these anxieties, none of the studies to date have shown that these concerns are substantially warranted, though ongoing surveillance and monitoring is essential. Although previous studies have suggested slow uptake of PrEP [39], more recent data suggest that there is increasing interest in PrEP in some urban centres where there is access to informed providers [40]. Clearly, optimal implementation of PrEP will require further refinements in both community and provider education. The challenges are daunting, but PrEP has the opportunity to be part of a response that can help arrest the continued spread of HIV in the United States and around the world.
